# Experimental Study on Prevention and Control of Calcium Carbonate Crystallization in Tunnel Based on the Yijun Tunnel

**DOI:** 10.1155/ianc/5977802

**Published:** 2025-10-13

**Authors:** Congnan Guo, Lijie Zhang, Guangxu Guo, Tianyong Wang, Yinyin Bai, Di Zhang, Tianzhi Yu

**Affiliations:** ^1^Xi'an Chengdu Railway Passenger Dedicated Line Shanxi Co., Ltd, Xi'an 710000, China; ^2^Lanzhou Jiaotong University, Lanzhou 730070, China; ^3^The 4th Engineering Co., Ltd. of China Railway 12th Bureau Group, Xi'an 710000, China

**Keywords:** blind pipe blockage, corrosion inhibition compound cleaning agent, crystallization dredging, crystallization mechanism, railway tunnel

## Abstract

The occurrence of crystallization plugging in the tunnel drainage system will lead to cracking and leakage of the tunnel lining. Therefore, it is very important to take effective measures to prevent the blockage of the crystallization pipe of the tunnel drainage system and ensure the safety and stability of the lining structure during the operation of the tunnel. In this study, we conducted field surveys and laboratory tests to analyze the relationship between the crystals in the Yijun tunnel blind pipeline and the concentration of various ions in the groundwater. X-ray diffraction (XRD) analysis confirmed that the main component of the crystalline sediment was calcium carbonate. The formation mechanism of crystallization was explored by using the environmental water chemical equilibrium software, Visual MINTEQ 3.1. Considering that the preparation of cleaning agent has less corrosive effect on tunnel concrete materials, the cleaning efficiency test of various acid cleaning agents on tunnel crystals was carried out to determine the optimal concentration of cleaning solution. Finally, it is determined that the mixture of formic acid: citric acid: corrosion inhibitor: surfactant: water = 1:0.5: 0.5:0.5: 5 is used as the cleaning agent of tunnel blind pipe. The experimental results show that the cleaning agent has a 92.4% CaCO_3_ dissolution rate, and the pH is controllable (4.7–7.9), which meets the environmental protection standards, and has little damage to the concrete (strength loss of 3.4 Mega Pascal, control group 6.2 Mega Pascal).

## 1. Introduction

With the rapid development of transportation infrastructure, a growing number of tunnel projects are being constructed and utilized, making tunnel construction an integral part of the modern transportation sector. However, after the construction of the tunnel, the original water system balance of the mountain was destroyed. The interaction between karst water and tunnel lining structure and drainage system makes the drainage system blocked by the precipitation and crystallization of rock debris wrapped by groundwater, which causes the drainage failure of the tunnel [1, 2]. The lining structure bears high external water pressure, endangers the safety of the tunnel structure, and even causes the tunnel structure to crack and produce water leakage disease, which causes great hidden dangers to the stability of the tunnel structure and vehicle driving [3]. According to the survey of railway tunnels, the workload of tunnel crystallization maintenance accounts for about 15%–20% of the total drainage facilities, while highway tunnels need to invest nearly 10,000 euros per kilometer for the maintenance of drainage systems every year [4, 5]. The prevention and treatment methods of drainage system blockage in karst tunnels have become a scientific and engineering problem to be solved urgently.

In order to solve the crystallization problem of the tunnel drainage system, scholars and engineers have done a lot of research on the causes, detection methods, and treatment measures of tunnel crystallization. At present, the coping strategies of tunnel blind pipe crystallization mainly include two aspects: prevention and treatment. Material optimization is the primary link to prevent crystallization blockage of the drainage system. Jiang et al. [6] significantly reduced crystal deposition by optimizing the hydrophobic coating to change the crystal phase and surface properties of calcium carbonate. Liu et al. [7] proposed that the flocked PVC tunnel drainage pipe can inhibit the adhesion of crystals on the pipe wall, and the use of a thermally conductive tunnel drainage pipe can avoid the crystallization blockage caused by the low temperature of the tunnel drainage pipe. In terms of treatment, cavitation jet technology has become the mainstream physical treatment method. By controlling parameters such as pressure and flow rate, a large number of cavitation bubbles are generated when the water beam passes through the cavitation nozzle. The cavitation bubble collapses in a narrow area on the surface of the material to produce a microjet impact of up to 140–170 Mega Pascal, so as to achieve the purpose of cleaning the surface attachments and fouling layer of the facility [8]. According to the analysis of crystal composition [9–12], Yoon et al. [13] proved that the main chemical composition of the crystal was CaCO_3_. Yang et al. [11] analyzed the effects of different parameters such as solution pH surface area and control saturation index (SI) and hydraulic conditions during the deposition of calcium carbonate in pipelines. In the study of using chemical methods to deal with crystallization, Ma et al. [14] prepared a solution to solve the blockage of the tunnel blind pipe cleaning agent solution. Hong et al. [15] determined the single-molecule carboxylic acid organic acid solvent for tunnel crystallization prevention. Liu et al. [16] analyzed the formation and treatment measures of tunnel crystals. At the same time, because the tunnel is mostly reinforced concrete structure, with the infiltration of water and chloride ions, the steel bar will be corroded, the corrosion products will be generated, and the expansion stress will be generated, resulting in the decrease of the stress structure function of the steel bar and the rupture and spalling of the concrete cover layer [17]. Finally, the concrete is destroyed and the building structure is at risk. At present, the cleaning agent suitable for crystallization dissolution of the tunnel drainage system is generally organic acid cleaning agent with weak acidity [18–21]. Although it can achieve better treatment effect, there are few reports on crystallization cleaning agent that solves crystallization and reduces concrete corrosion [22, 23].

Based on the Yijun tunnel project, this paper proposes a chemical treatment method to remove the crystallization of the tunnel drainage blind pipe. The physical structure and chemical composition of the crystals were obtained by tunnel field sampling test, X-ray diffraction, and chemical analysis test. The precipitation formation mechanism of Ca^2+^ ions with pH change in environmental water was analyzed by using the environmental water chemical equilibrium software, Visual MINTEQ 3.1. Then, taking into account the influence of the preparation of cleaning agents on the corrosion of tunnel concrete materials, by comparing the cleaning efficiency of single acid, mixed acid, and corrosion inhibitor compound, considering the corrosion of acid on concrete, it is suggested to use the corrosion inhibitor compound as a cleaning agent for the tunnel blind pipe and describe the recommended ratio. Finally, the relationship between the cleaning agent and the dissolution rate and pH was analyzed, and it was verified that the compound cleaning agent had a certain corrosion inhibition effect on the concrete material.

## 2. Experimental Materials and Instruments

Tunnel rock samples, crystalline solid samples, and water samples were collected from the Yijun Tunnel prepared by the 12th Bureau of China Railway. Cement, fly ash, water-reducing agent, accelerator, and other building materials are provided by China Railway 12th Bureau.

Reagents, such as nitric acid (HNO_3_), hydrochloric acid (HCl), hydrogen peroxide (H_2_O_2_), oxalic acid, citric acid, and Xi'an Chemical Reagent Factory, are analytically pure, acetate fiber filter membrane (0.45 μm), Tianjin Tiangan Chemical Technology Development Co., Ltd. Among them, nitric acid, hydrogen peroxide, and acetate fiber filter membrane are used in the digestion process of water sample treatment, hydrochloric acid, oxalic acid, and citric acid, which are used in the preparation of tunnel crystallization cleaning agent.

**Figure 1:**
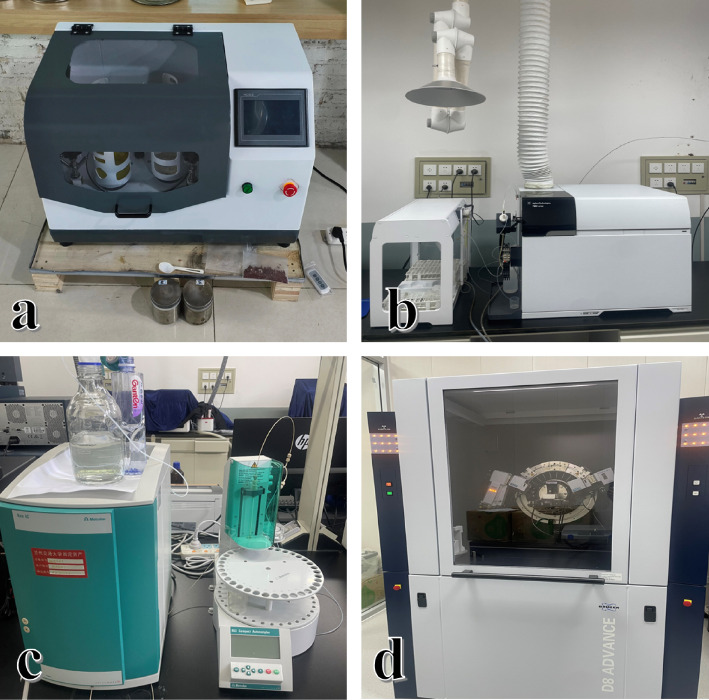
Laboratory equipment. (a) DECO light vertical planetary ball mill; (b) Agilent 7900 Inductively Coupled Plasma Emission Spectrometer; (c) ECO-IC Ion Chromatograph; (d) BRUCKER D8 ADVANCE X-ray diffractometer.

Instruments (Figure 1) used were as follows: BSA224S electronic balance, Beijing Sedolis Scientific Instrument Co., Ltd (For weighing samples); J-1 constant speed electric stirrer, Zhengzhou Dufu Instrument Factory (for mixed reagents); HH-2 digital electric thermostatic water bath, Beijing Kewei Yongxing Instrument Co., Ltd. (for digestion of water samples); DZF-6020 vacuum drying oven, Shanghai Hengke Instrument Co., Ltd. (for drying filtered crystals); DECO light vertical planetary ball mill, Changsha Deke Equipment Co., Ltd. (for crushing solid sample); ECO-IC Ion Chromatograph, Metrohm (for ion detection and analysis in water samples); Agilent5110 Inductively Coupled Plasma Emission Spectrometer, Agilent Technology Company (For elemental analysis in water samples); and BRUCKER D8 ADVANCE X-ray diffractometer, Bruker (for identify the crystal structure).

## 3. Results and Discussion

### 3.1. Distribution Characteristics of Tunnel Crystals

**Figure 2:**
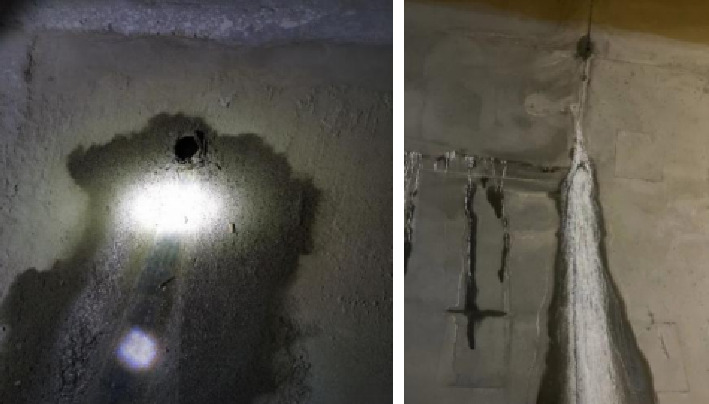
Crystal and white precipitate of blind pipe.

Figure 2 shows the crystal and water samples collected at the entrance and exit of Yijun Tunnel. There was obvious white crystal precipitation in the tunnel blind pipe, and more crystallization accumulated in the blind pipe mouth. The effluent water sample is a clear and transparent liquid after static precipitation.

### 3.2. Water Sample Analysis of Blind Pipe in Xiyan High-Speed Railway Tunnel

#### 3.2.1. Tunnel Water Sample Data Summary

The contents of Na^+^, Ca^2+^, Mg^2+^, CO_3_^2−^, HCO_3_^−^, and SO_4_^2−^ in the surrounding rock water, outer layer seepage of shotcrete, blind drainage, cement leachate, and fly ash leachate were determined and named as Samples 1, 2, 3, 4, and 5, respectively.  Table 1 summarizes the data from the analysis of water samples in the Yijun Tunnel. According to the data, it is inferred that calcium carbonate and magnesium carbonate are the main components of the crystalline sediments that cause the blockage of the blind pipe of the Yijun Tunnel [24]. At the same time, the ability of carbonate to bind hydrogen ions and calcium ions is stronger than that of sulfate, and the Ksp of calcium carbonate is smaller than that of calcium sulfate. At the same temperature, the solubility of calcium carbonate is less than that of calcium sulfate, so carbonate is more easily consumed to form calcium carbonate precipitation, resulting in a higher concentration of sulfate ions than carbonate ions.

#### 3.2.2. Analysis of the Influence of Groundwater Flowing Through Tunnel Surrounding Rock on Crystallization

**Figure 3:**
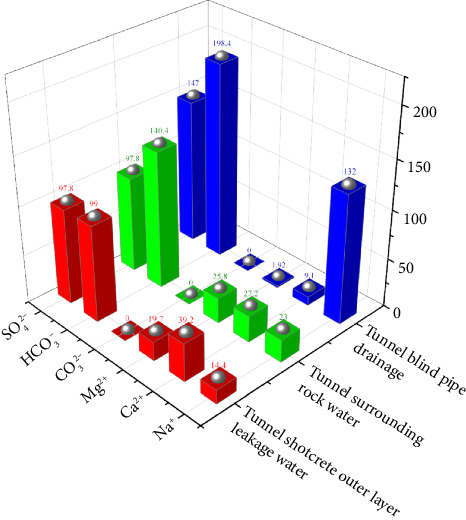
The ion concentration of tunnel surrounding rock water, tunnel shotcrete outer layer leakage water, and blind pipe drainage.

Figure 3 summarizes the ion concentration of each water sample in the tunnel surrounding rock water, the tunnel shotcrete outer layer leakage water, and the tunnel blind pipe drainage. As can be seen from Figure 3, the anions in tunnel surrounding rock water and the outer layer of tunnel shotcrete seepage and blind pipe drainage are mainly sodium bicarbonate and sulfate ions, and the main cations are Ca^2+^, Mg^2+^, and Na^+^ ions. When the water flows through the surrounding rock formation of the tunnel, the Ca^2+^ concentration increased significantly, indicating that the groundwater flowing through the tunnel dissolved some inorganic salts from the rock formation. The concentration of calcium ions in the water sample seeping from the blind pipe after concrete is significantly reduced, and a large number of white crystals are precipitated on the blind pipe mouth and the tunnel wall, indicating that the inorganic salts dissolved in the rock layer are the main source of Ca^2+^ and Na^+^ in the crystals, and the ions that can cause crystallization in the building materials cannot be ignored.

### 3.3. Analysis of Crystal Solid Sample of Blind Pipe in Yijun Tunnel

#### 3.3.1. Analysis of Crystal Composition in Tunnel Blind Pipe

**Figure 4:**
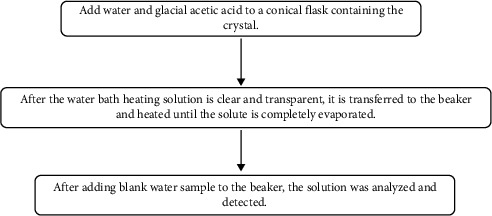
Crystal processing and ion content detection process.

In order to formulate a solvent specifically targeting the identified crystalline substances, it is crucial to accurately determine the composition of the crystals. Based on the preliminary inference from the water sample analysis, the main components of the crystals in the Yijun Tunnel are carbonate minerals such as calcium carbonate and magnesium carbonate. Therefore, a sample of 3-g tunnel crystalline sediment was put into a conical flask, 20 mL of water, and 20 mL of glacial acetic acid were added to the flask successively, and the solid sample was stirred until dissolved to remove carbonate ions. Dissolve part of the insoluble solid by heating in the water bath, transfer the solution to the beaker after the solution becomes transparent, and heat with the heating furnace until the solvent is totally volatilized. The solute is collected, and the resulting solid sample is dried. At this stage, the solid sample mostly contains soluble salts. The solid sample was dissolved in water and the aqueous solution was further analyzed, and the data are attributed to Table 2. The data show that the dominant ions in the crystalline deposits are Ca^2+^ and Cl^−^. The overall process is shown in Figure 4.

#### 3.3.2. X-Ray Diffraction of Crystals

**Figure 5:**
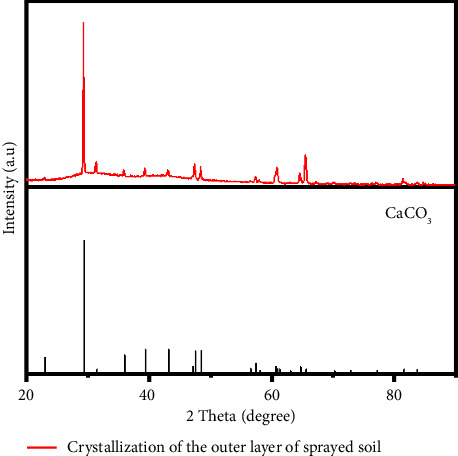
Crystalline XRD pattern and standard calcium carbonate XRD pattern.

The diffraction angle in Figure 5 refers to the angle between the direction of the diffraction beam and the direction of the incident beam after the incident beam is reflected and diffracted at the edge of the crystal. The intensity in the figure refers to the intensity of the X-ray after the incident beam completes the diffraction. The XRD peak shape of the sample to be tested is sharp and prominent, reaching the peak at 2*θ* = 29°, which is highly coincident with the calcium carbonate CaCO_3_ standard card. XRD results show that the main component of the crystal is calcium carbonate. This conclusion is consistent with the research results of Chen et al. [25]. In addition, although the concentration of sulfate ions in the water sample analysis is higher than that of carbonate ions, the characteristic peak of calcium sulfate (2*θ* = 25.6°) only accounts for 3.2%, and the solubility product of calcium carbonate (Ksp = 3.36 × 10) is significantly lower than that of calcium sulfate (Ksp = 4.93 × 10). This order of magnitude difference makes it easier for calcium carbonate to reach supersaturation and precipitate under the same ion concentration conditions.

### 3.4. Simulation Analysis of Crystallization Process

**Figure 6:**
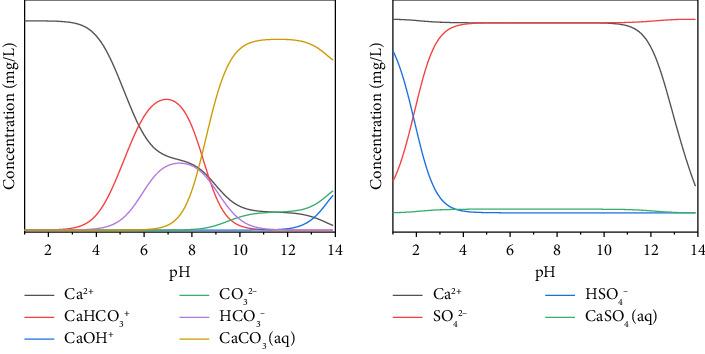
Ion distribution map of Ca^2+^ ion aqueous solution (a) CaCO_3_ and (b) CaSO_4_.

**Figure 7:**
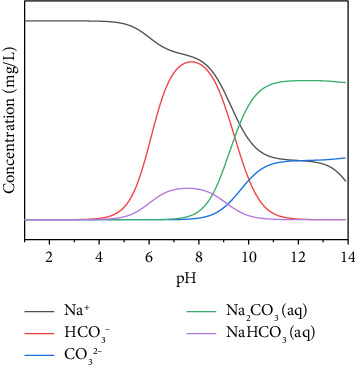
The ion distribution diagram of each component of sodium ion aqueous solution.

In this paper, the precipitation mechanism of Ca^2+^ and Na^+^ ions in water with pH shift was analyzed by using the environmental water chemical equilibrium software Visual MINTEQ 3.1. The initial pH value was set to 1, the ionic strength value was set to 0.001, the concentration of calcium ion and sodium ion in the tunnel blind tube water sample is the initial condition, and the unit was unified to mg/L. As shown in Figure 6, first the distribution of Ca^2+^ ions with carbonate and sulfate at different pHs is calculated. It can be concluded that carbonate (CO_2_) contributes further to the precipitation, which is consistent with the test results of crystal precipitation samples. The main precipitation component is CaCO_3_.  Figure 7 shows the ionic distribution of each component of the aqueous solution of Na^+^ ions. Due to its large solubility, it forms a mixed precipitate with CaCO_3_ upon supersaturation. Therefore, the software simulates the precipitation process of CaCO_3_. CO_2_ dissolves in water to form carbonic acid.

The equation for the ionization reaction is as follows:

(1) $$\begin{array}{c} {\text{CO}}_{2}+H_{2}O⇌H_{2}{\text{CO}}_{3} \end{array}$$

(2) $$\begin{array}{c} H_{2}{\text{CO}}_{3}⇌H^{+}+{\text{HCO}}_{3}^{-} \end{array}$$

(3) $$\begin{array}{c} {\text{HCO}}_{3}^{-}⇌H^{+}+{\text{CO}}_{3}^{2-} \end{array}$$

**Figure 8:**
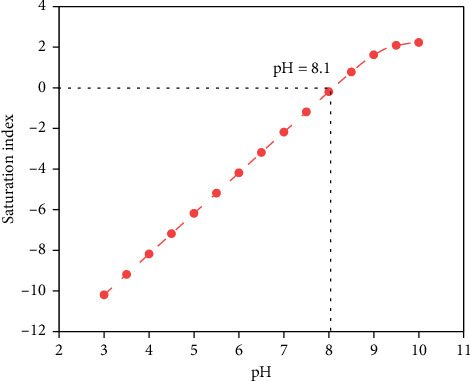
Saturation index curve of CaCO_3_ with pH value.

The concentrations of ions in the tunnel surrounding rock water are from high to low: HCO_3_^−^ > Ca^2+^ > CO_3_^2−^. Among them, the HCO_3_^−^ content is relatively high, at 214.3 mg/L. The calcium ion concentration is relatively high, and the CO_3_^2−^ ion concentration does not reach the detection limit, indicating that the crystals precipitated in the karst tunnel blind pipe are mainly produced by the precipitation of crystals generated by chemical reactions under the condition of changes in solubility. The ratio of the concentrations of the three types of carbonic acid in water at equilibrium shows a complete correspondence with the pH value. In the low pH range, only HCO_3_^−^ + CO_2_ is present in the water. There are only CO_3_^2−^ ions in the extreme pH range. HCO_3_^−^ dominates in the range of moderate pH values. As a result, the precipitation of calcium carbonate increases when the pH of the water is high; conversely, when the pH of water is low, calcium carbonate does not precipitate easily. The pH value has a great influence on the solubility of carbonates, and reducing the pH value will increase their solubility. As shown in Figure 8, when the pH is between 3 and 8, the precipitate is not easy to form; when the pH is between 8 and 10, the precipitate formation rate is significantly increased; and when the pH is greater than 10, the precipitate formation rate tends to be flat. It can be seen that the value of pH has a large effect on the solubility of calcium carbonate precipitates. Real-time monitoring of water pH can improve tunneling crystallization to some extent.

**Figure 9:**
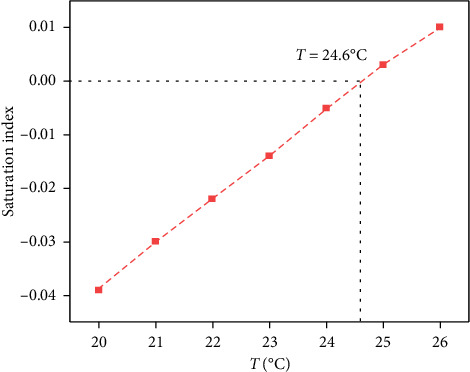
Saturation index curve of CaCO_3_ with temperature.

**Figure 10:**
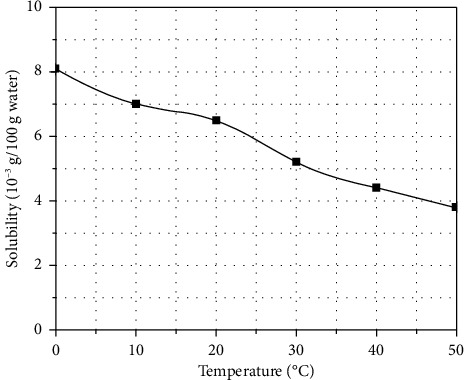
The solubility curve of CaCO_3_ varies with temperature.

Run the model program to calculate. Visual MINTEQ will iterate repeatedly based on the initial concentration of the basic components, the dissolution/precipitation equilibrium reaction equation and the equilibrium constant of the substances in the system, and simulate the chemical form (molecular or ionic composition) and concentration of each substance in the solution when the calculation system reaches equilibrium. Visual MINTEQ will automatically calculate the SI of each substance. The SI value can be used to determine whether the substance is likely to precipitate. When SI < 0, it means that the concentration of the substance in the solution does not exceed its solubility and will not precipitate; when SI > 0, the substance is oversaturated and will precipitate. At this time, all substances with SI > 0 will precipitate, and the amount of precipitation will continue to participate in the iteration as a new variable until the substance SI = 0; that is, the dissolution/precipitation equilibrium is reached. After the calculation is completed, the solution equilibrium calculation results (including the concentration and chemical form of dissolved substances, the chemical form of precipitated substances, the pH at equilibrium, and the SI of substances) are extracted in combination with the output file given by the program. The data were summarized in Origin, and the trend curve of the SI of CaCO_3_ in an open system with temperature at pH = 8.1 and a temperature range of 20.0–26.0°C was drawn based on the convergence data (Figure 9). As the temperature increases, the SI of CaCO_3_ increases, and the critical temperature for CaCO_3_ precipitation under this condition is about 25.0°C.  Figure 10 shows the solubility curve of CaCO_3_ with temperature. Temperature is another important factor affecting the precipitation of calcium carbonate. The solubility of most salts in water increases with the increase in temperature. However, calcium carbonate has an abnormal solubility, and the solubility decreases when the temperature increases, that is, more calcium carbonate will be precipitated when the water temperature increases.

The study explored the mechanism of crystallization blockage in Yijun Tunnel through software simulation experiments of the crystallization process and ion concentration analysis of tunnel water samples. The results indicate that the formation of carbonate crystallization precipitation is related to the ion concentration, pH value, temperature, pressure, and solution properties of the solution, with the crystallization process being the combined effect of many factors.

During tunnel excavation, the partial pressure of CO_2_ gas in groundwater is reduced, causing dissolved CO_2_ to escape and decreasing the concentration of H^+^, which promotes the chemical reaction towards precipitated crystal formation. Dissolved calcium, magnesium, and sodium ions in groundwater flowing through the surrounding rock precipitate as carbonates. Additionally, alkaline substances like primary support shotcrete dissolve in the surrounding rock's fissure water, making the water strongly alkaline and significantly increasing calcium and magnesium ion content. In low water flow rate conditions, carbonate has more time to crystallize, making the drainage system more prone to clogging. From the above analysis, the scaling ions mainly originate from two sources: the groundwater itself (interacting with rock, etc.) and the dissolution of concrete. The tunnel surrounding rock's groundwater contains various soluble salts, such as bicarbonate, sulfate, phosphate, chloride, soluble carbonate, and silicate. The tunnel shotcrete is rich in calcium salts, and groundwater seepage brings calcium ions into the tunnel blind pipe through the shotcrete. When in contact with air, a large amount of CO_2_ dissolves in water, resulting in high concentrations of CO_3_^2−^ and HCO_3_^−^ in groundwater. HCO_3_^−^ is unstable and easily decomposes to form CO_3_^2−^. The combination of Ca^2+^ and CO_3_^2−^ readily forms insoluble calcium carbonate. Calcium carbonate crystals precipitate from water and slowly grow on the inner wall of the blind pipe. Cement and fly ash contain a large amount of silicon dioxide, and the combined effects of these factors ultimately lead to blind pipe crystallization.

### 3.5. Comparison of Crystal Treatment Schemes

Combined with the mechanism of tunnel crystallization, three different chemical dissolution methods, single acid method, mixed acid method, and comprehensive compound method, were used to treat tunnel crystallization in this study. Through the detailed analysis and comparative study of dissolution time, reaction conditions, and dissolution effects, the most suitable dissolving agent for solving the problem of blind pipe blockage was finally selected.

#### 3.5.1. Single Acid Method

After comparing and analyzing the experimental phenomena, a single acid-dissolving agent was used for testing, and the test results are listed in Table 3. From the screening results, although hydrochloric acid has a good dissolution effect, its corrosiveness is too strong, so it is not recommended to be used independently. Formic acid shows a good cleaning effect and can be regarded as an independent cleaning agent. However, acetic acid or citric acid was used as the cleaning agent alone, the reaction rate was relatively slow, so not suitable alone. In addition, the acid-dissolving agent itself has a certain degree of corrosion, which may adversely affect the main structure of the tunnel. Therefore, it is recommended to use it in combination with other reagents to reduce its corrosion.

#### 3.5.2. Mixed Acid Method

According to the results of Table 1, the independent use of a single weak acid as a solvent failed to meet the standards required for the test. Therefore, we tried to mix the two acids in a certain proportion to test their dissolution effect on the crystal. The specific test results are shown in Table 4.

It can be seen from the data in Table 4 that the dissolution rate of tunnel crystallization is significantly improved when the strong acid and weak acid are mixed as the reaction liquid. This is because hydrochloric acid has strong acidity. After adding hydrochloric acid, it first reacts with tunnel crystallization, and formic acid and acetic acid provide additional hydrogen ions, which promotes the continuous dissolution of crystals. The reaction rate of the reaction solution of mixed formic acid and citric acid is slightly slower at room temperature, but the dissolution effect is better. Although the mixed acid can be used to remove the crystals blocked by the tunnel, the acidic dissolution agent may have an adverse effect on the tunnel structure, so it is recommended to be used in combination with other corrosion inhibitors.

#### 3.5.3. Corrosion Inhibitor Compounding Method

Through the experimental results of Tables 3 and 4, it is found that the treatment effect of mixed acid on tunnel crystallization is stronger than that of single acid, but it still poses a threat to the strength and durability of tunnel lining concrete structure and drainage system materials. The corrosion of metals is mostly the result of the galvanic cell reaction on the metal surface (the galvanic cell reaction includes the anode reaction and the cathode reaction), which is also the most important factor causing the corrosion. The reaction group on the inhibitor molecule interacts with the metal ions generated during the corrosion process to form a precipitation film, which inhibits the electrochemical process of the anode and cathode. Therefore, on the basis of transforming crystals into water-soluble metal ions that are easily free by mixed acid method, it is compounded with corrosion inhibitors that have a slow-release effect on metals and cement to reduce the corrosion of tunnel walls. The interaction between the pickling agent and the alkaline substance in the cement stone reduces the alkalinity of the concrete, and the passivation film around the steel bar is destroyed. After the corrosion and expansion of the steel bar, the concrete cover will crack and peel off. At the same time, concrete is more prone to carbonation to aggravate the shrinkage of concrete, which may lead to cracks in concrete and structural damage. The introduction of corrosion inhibitor can inhibit the ability of steel to absorb hydrogen during pickling, avoid “hydrogen embrittlement” of steel, and inhibit the corrosion of Fe^3+^ to metal during pickling, so that the metal does not produce pitting corrosion. At the same time, surfactants and thickeners are added to improve the lubricity of the liquid and the inner surface of the container, and the adhesion of the precipitate to the pipeline is reduced. Finally, the dissolved loose attachment is washed out of the pipeline by water pressure to reach the cleaning effect. The specific compounding scheme is as follows:

  Scheme (1). Formic acid: Citric acid: Water = 1:0.5:5 (mass ratio)
  Scheme (2) Formic acid: Citric acid: Corrosion inhibitor: Water = 1:0.5:0.5:5 (mass ratio);
  Scheme (3) Formic acid: Citric acid: Corrosion inhibitor: Surfactant: Water = 1:0.5:0.5:0.5:5 (mass ratio);
  Scheme (4). Formic acid: Citric acid: Corrosion inhibitor: Surfactant: Water = 2:1:0.5:0.5:2 (mass ratio);
  Scheme (5). Distilled water.

**Figure 11:**
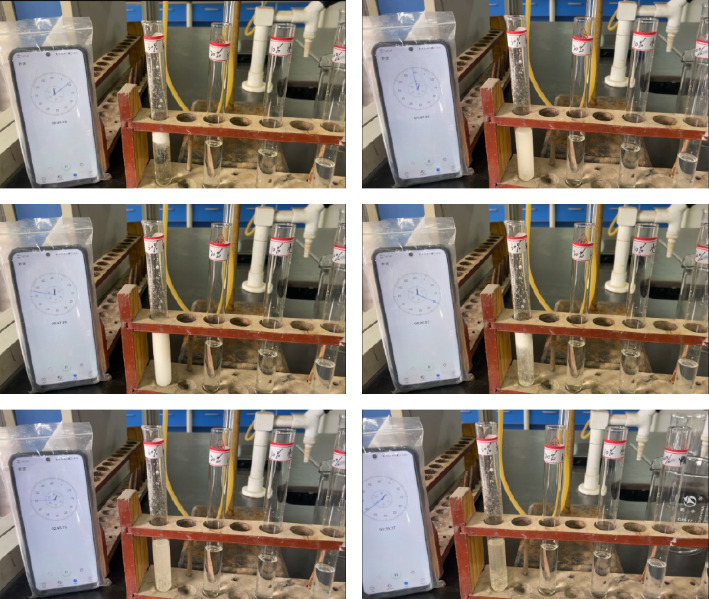
Dissolution test process.

In the experiment, 1-g white crystal was placed in different solvents to observe the experimental phenomenon, and the most suitable solvent was selected through a series of comparative experiments. The experimental rules and test results are recorded in Table 5. It was found that formic acid, citric acid, and the solution containing proper amount of corrosion inhibitor and thickening lubricant (accounting for 10% of the dissolved liquid volume) had a good dissolution effect on the tunnel crystal at room temperature (Figure 11).

The dissolution effect of tunnel crystals is affected by many factors, such as the type of organic acid, the concentration of organic acid solution, the flow rate of organic acid solution, the temperature, the pH value of the solution, the surface area of the crystal, and so on. However, from an intuitive point of view, the chemical dissolution process of the tunnel crystal is that calcium carbonate continuously dissolves and peels off the crystal, and the crystal is finally disintegrated. Therefore, the removal effect of the tunnel crystal in the tunnel drainage system can be calculated by the determination of the calcium ion content in the solution in the tunnel crystal dissolution test to calculate the dissolution rate, so as to compare the cleaning effect of different types of organic acids and their corresponding concentrations.

**Figure 12:**
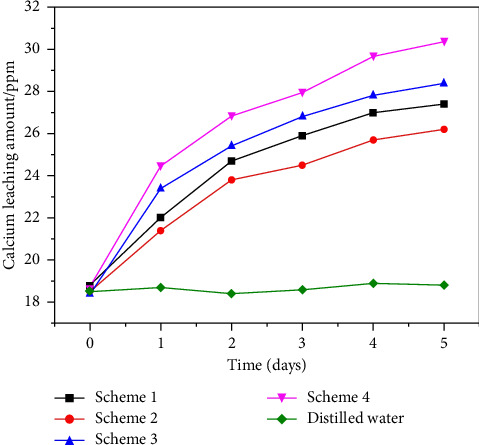
Calcium content of five schemes under different soaking times.

Figure 12 shows the change of calcium ion content in the four different schemes of tunnel crystal block soaking for different times (the data are recorded in Table 6). Since the hydrogen ions in distilled water will undergo a slight ionization reaction with CaCO_3_, the surface ionization of the crystal will soon terminate, so the calcium ion content will be maintained at 18.6 ppm. In the four schemes, with the increase of soaking time, the concentration of Ca^2+^ increased gradually. Compared with Scheme 1 and Scheme 2, the acidity of Scheme 3 and Scheme 4 is stronger, and the dissolution of Ca^2+^ is larger. Among them, the concentration of acid in Scheme 4 is larger, resulting in more Ca^2+^ dissolution and complete dissolution of crystals. However, since Scheme 4 may be corrosive to cement, it is not an ideal cleaning agent. In contrast, the effect of dissolving crystals in Scheme 3 is better, and it will not cause damage to the tunnel structure. Therefore, Scheme 3 is an ideal cleaning agent.

**Figure 13:**
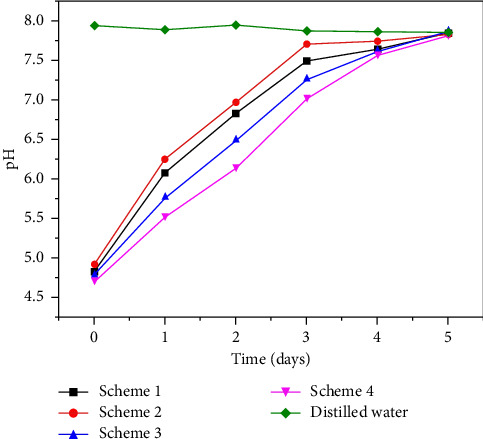
pH values of five solutions at different soaking times.

In the tunnel crystal dissolution test, in order to evaluate the impact of the waste liquid discharged after cleaning the crystal on the water environment, the pH value of the soaking liquid was measured regularly. Every 24 h, a pH meter is used to directly measure the pH value of the soaking solution in the beaker, and the data (Table 7) are recorded after the reading is stable. As shown in the figure, the pH changes of the soaking solution in the four different schemes can be seen. The soaking solution of the crystal sample soaked in distilled water is neutral, and the pH value fluctuates slightly between 7.8 and 8.0. However, the pH value of the crystal samples soaked in four different schemes showed a downward trend over time. As shown in the table, the range of pH value is between 4.7 and 7.9 (Figure 13). It is worth noting that the pH values of all soaking solutions are within the allowable range of the environment. These results show that when cleaning the tunnel crystal blockage in the tunnel drainage system, the selected cleaning scheme can ensure that the pH value of the waste liquid will not adversely affect the water environment while ensuring the cleaning effect. However, continuous monitoring is still necessary to ensure long-term environmental effects.

#### 3.5.4. Dissolution Rate Test of Crystals

The dissolution rate of tunnel crystals is affected by different acid types, acid concentration, solution pH value, and ambient temperature. However, from an intuitive point of view, the chemical dissolution process of tunnel crystals is the process of continuous dissolution and stripping of calcium carbonate on the surface of the crystal and complete decomposition of the final crystal. Therefore, the dissolution rate of tunnel crystals in acid cleaning agent can be characterized by the change of crystal mass in the crystal dissolution experiment.

**Figure 14:**
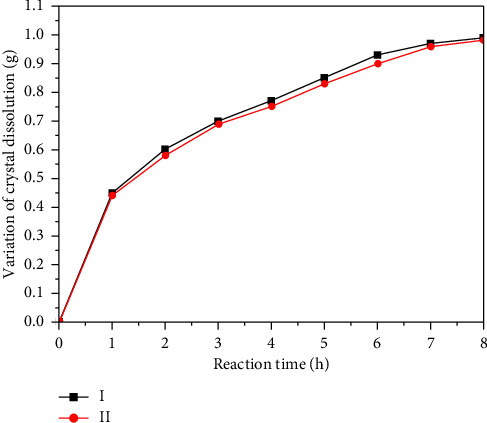
The cumulative change curve of crystal sample mass.

Figure 14 shows the curve of the cumulative change in the mass of the tunnel crystal sample in the cleaning agent under different conditions (Curve I represents no corrosion inhibitor cleaning agent; Curve II represents the addition of corrosion inhibitor cleaning agent). The diagram visually reflects the difference in the mass change during crystal dissolution with and without corrosion inhibitor in two curves (Curve I and Curve II). The Curve I in Figure 14 (cleaner without corrosion inhibitor) depicts the trend of crystalline sample quality over time when no corrosion inhibitor is added to the cleaner. In the initial stage of the dissolution experiment, especially in the first 1 h, the crystal quality decreases sharply, indicating that the reaction between the cleaning agent and the tunnel crystal is the most violent, and the dissolution rate is the fastest. Subsequently, as the reaction progressed, the dissolution rate gradually slowed down until after about 5 h, when the curve leveled off, indicating that the dissolution rate of calcium carbonate became extremely slow or almost stopped, and the dissolution process was almost over. Curve II (cleaning agent with corrosion inhibitor) in Figure 14 compared with the Curve I, this curve shows a small decrease in crystal quality during dissolution and a slightly smaller change in mass during dissolution, reflecting the slow-release effect of the cleaning agent on crystal dissolution when corrosion inhibitor is added. From the perspective of the curve trend, the solubility of the cleaning agent with corrosion inhibitor is relatively weak, which is conducive to the protection of building materials.

**Figure 15:**
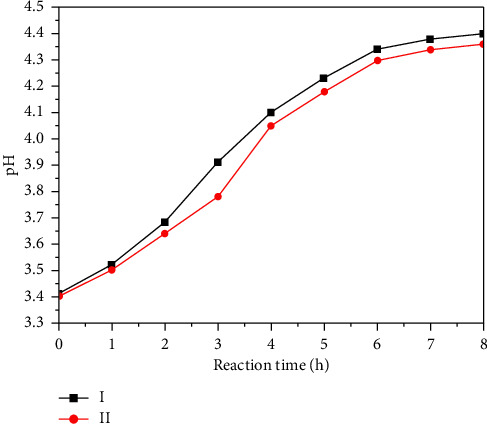
The pH change curve of cleaning agent with reaction.

Figure 15 shows the pH of the cleaning agent during the reaction, and two curves (I and II) show the pH of the cleaning agent solution with and without corrosion inhibitors (Curve I represents the addition of corrosion inhibitor cleaning agent; Curve II represents no corrosion inhibitor cleaning agent). The Curve I in Figure 15 (with the addition of corrosion inhibitor) shows that the pH of the solution decreases rapidly at the beginning of the dissolution experiment due to the rapid reaction of the cleaning agent with the tunnel crystals. When the pH drops to a certain level (pH = 4), the rate of decline slows down, indicating that the reaction rate or dissolution efficiency begins to decrease. As the reaction continued, the pH value change tended to be stable, reflecting the consumption of acidic substances in the cleaning agent and the establishment of reaction equilibrium. The Curve II in Figure 15 (cleaner without corrosion inhibitors) shows a sharp drop in pH of the cleaner solution at the initial stage and then gradually stabilizes. In the absence of corrosion inhibitors, the change in pH value is more direct and significant, because the presence of corrosion inhibitors buffers the change in pH value to a certain extent, which further confirms that the corrosion inhibitor has a significant slow-release effect on the final pH value of the cleaning agent. Figures 14 and 15 jointly reveal the dissolution process of tunnel crystals in acid cleaning agent and the characteristics of crystal dissolution degree before and after the addition of corrosion inhibitors, which provides an important reference for optimizing the cleaning agent formulation and improving the cleaning efficiency.

#### 3.5.5. Compressive Strength Test

**Figure 16:**
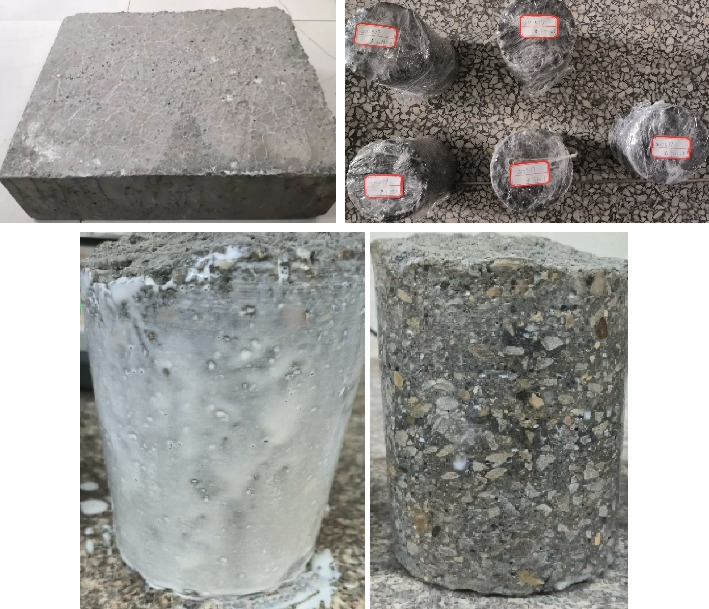
Experimental samples of concrete.

Because the cleaning solution prepared by removing the crystallization of the blind pipe contains mixed acid, in order to ensure that there is no corrosive damage to the concrete products in the tunnel wall, it is necessary to carry out corrosive test on the blind pipe crystallization solution. For the DK139 + 190 section of the Yijun Tunnel of the 12th Bureau of China Railway, a 5-cm-thick test block was cut from the surrounding rock (inside). As shown in Figure 16, the large-scale concrete samples were drilled and sampled. The size of the large-scale sample is 35 × 40 × 12 cm, the diameter of the core sample is 100 mm, and the height is 120 mm, a total of three. The side of the drilled core sample is coated with epoxy resin. The waterborne epoxy resin and the waterborne epoxy curing agent are mixed at a ratio of 2:1. The mixture with a total weight of about 100 g needs to be stirred at one time. The application was carried out in two times. After the first application, the second application was carried out at an interval of 1 hour. After the completion of the application, the mixture was milky white, and it was allowed to stand at room temperature for at least 24 h until the milky white epoxy resin became transparent and completely solidified (it was smooth and nonsticky by hand touch).

**Figure 17:**
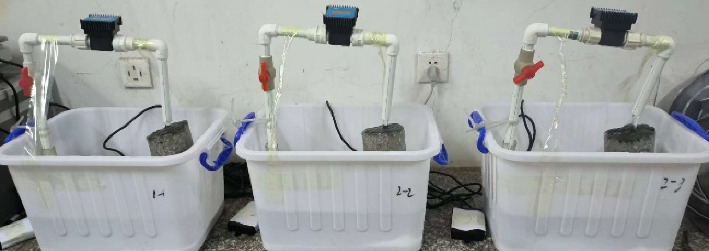
Immersion experiment of concrete.

**Figure 18:**
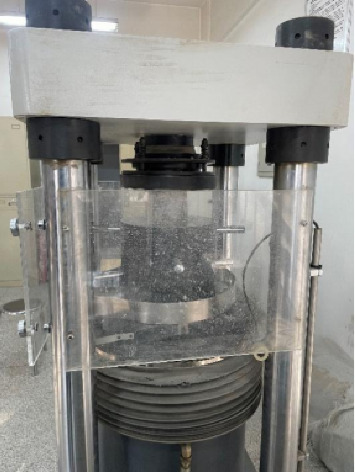
Concrete compressive strength experiment.

**Figure 19:**
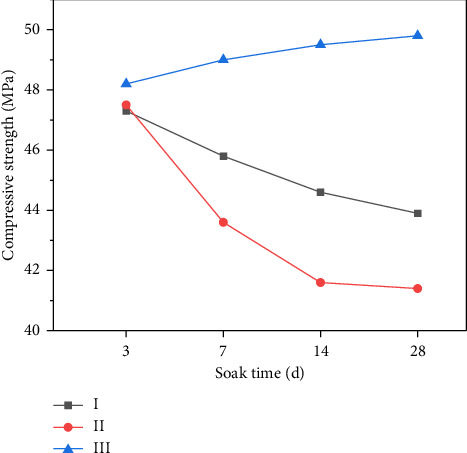
The change curve of compressive strength of concrete with soaking time.

As shown in Figure 17, the concrete samples of Yijun tunnel were immersed in cleaning agent with and without corrosion inhibitor and distilled water, respectively. The compressive strength was measured after soaking for 3, 7, 14, and 28 days.  Figure 18 documents the compressive strength testing methodology. The resultant strength curves in Figure 18 compare three conditions: (I) inhibitor-modified cleaning solution, (II) base cleaning solution, and (III) distilled water control. From Figure 19, it can be seen that the reference group immersed in distilled water generates more C-S-H gel due to hydration to enhance the strength of the matrix, and the 28-day immersion increases the strength by 1.2 Mega Pascal (2.5% increase). The compressive strength of concrete specimens immersed in the cleaning agent not compounded with the corrosion inhibitor decreases with the increase of immersion time. From the third day of soaking to the 28th day of soaking, the compressive strength decreases from 47.5 Mega Pascal to 41.3 Mega Pascal, and the change value of compressive strength is 6.2 Mega Pascal. The compressive strength change of the concrete test block soaked in the compound corrosion inhibitor cleaning agent is 3.4 Mega Pascal. It can be seen that the cleaning agent with corrosion inhibitor can reduce the corrosion of concrete while preventing the crystallization of the tunnel.

## 4. Conclusions

Based on the Yijun tunnel project, this paper proposes a chemical treatment method to remove the crystallization of the tunnel drainage blind pipe. Through the tunnel field sampling test, X-ray diffraction, and chemical analysis test, it is proved that the crystal of the tunnel blind pipe is mainly calcium carbonate. The effects of pH and temperature on the precipitation of Ca^2+^ ions in environmental water were analyzed by Visual MINTEQ 3.1. Then, considering the influence of the preparation of the cleaning agent on the corrosion of the tunnel concrete material, by comparing the cleaning efficiency of single acid, mixed acid, and corrosion inhibitor compound, and considering the corrosion of acid on concrete, it is suggested to use corrosion inhibitor compound as cleaning agent for tunnel blind pipe. Using formic acid: citric acid: corrosion inhibitor: surfactant: water = 1:0.5: 0.5:0.5: 5 ratio, the dissolution efficiency of Ca^2+^ reached 28.4 ppm in five days. The pH is maintained in an environmentally safe range of 4.7–7.9. The compressive strength loss of concrete was only reduced by 3.4 Mega Pascal (6.2 Mega Pascal in the control group). It is verified that the composite cleaning agent has a certain corrosion inhibition effect on concrete materials.

## Figures and Tables

**Table 1:** Water quality analysis of samples (mg/L) ions.

Parameters	Sample 1	Sample 2	Sample 3	Sample 4	Sample 5
Ca^2+^	27.2	39.2	9.10	373	293
Mg^2+^	25.8	19.7	1.92	0.622	0.501
Na^+^	23.0	14.4	132	117	50.6
CO_3_^2−^	No detection	No detection	No detection	No detection	No detection
HCO_3_^−^	140.4	99.0	198.4	140.7	80.5
SO_4_^2−^	97.8	97.8	147	1590	530

**Table 2:** Analysis of crystalline deposits from the Yijun tunnel.

Crystal name	Ion content (mg/L)				
	Na^+^	Ca^2+^	Mg^2+^	K^+^	Cl^−^
The Yijun Tunnel blind pipe crystallization	0.21	161	0.87	0.03	60.457

**Table 3:** Treatment effect of single acid method.

Reagent selection^a^	Concentration	Extent of reaction	Solution time (min)	Degree of dissolution	Corrosiveness	Applicability analysis
Hydrochloric acid	100%	Violent reaction	10	Complete dissolution	Strong	The effect is obvious but the corrosion is strong, so it is not recommended to use alone
	50%	Violent reaction	15	Complete dissolution	Strong	
	30%	Fast reaction	17	Complete dissolution	Strong	

Formic acid	100%	Fast reaction	12	Complete dissolution	Relatively strong	Good applicability and moderate corrosivity can be selected as a cleaning agent
	50%	Fast reaction	16	Mostly dissolved	Relatively strong	
	30%	Moderate reaction	18	Half dissolved	Moderate	

Acetic acid	100%	Fast reaction	16	Mostly dissolved	Moderate	Poor solubility, moderate activity, and certain corrosiveness are not recommended for use alone
	50%	Moderate reaction	19	Half dissolved	Moderate	
	30%	Moderate reaction	25	Partial solution	Weak	

Citric acid	100%	Moderate reaction	18	Partial solution	Weak	The activity is poor, but the corrosivity is extremely weak, so it is recommended to mix with other acids
	50%	Moderate reaction	22	Partial solution	Weak	
	30%	Slow	27	Partial solution	Weak	

^a^All solvent tests were carried out at 25°C with a solvent volume of 10 mL.

**Table 4:** Treatment effect of mixed acid method.

Mixed acid ratio (1:4)	Experimental phenomena	Experimental conclusion
Hydrochloric acid + formic acid	The reaction rate is obvious, a large number of bubbles are generated, and the dissolution is faster	Strong corrosion may affect the tunnel
Hydrochloric acid + acetic acid	The reaction is fast, the solid dissolution rate is moderate and a large amount dissolved	Strong corrosion may affect the tunnel
Citric acid + formic acid	The reaction is relatively moderate, the solid is slowly dissolved, and the amount of dissolution is acceptable	Moderate corrosivity and reaction rate
Citric acid + acetic acid	The reaction is slow, the dissolution rate is low, and most of the solid is not dissolved	The corrosion is weak, but the reaction rate is slow

**Table 5:** Treatment effect of corrosion inhibitor compound method.

Scheme	Reaction phenomenon	Conclusion of the experiment
(1)	Bubble formation, complete dissolution, slow speed	Dissolution is acceptable, but it is corrosive to cement
(2)	There is a gas release, speed before and after slow, dissolved completely	Dissolution is acceptable, but the rate is slow
(3)	The reaction is fast and the dissolution effect is good	Good dissolution effect, moderate reaction, can be used as a reaction solution
(4)	Strong reaction, there are bubbles released, dissolved completely	The dissolution effect is good, but the reaction is violent and may corrode to the tunnel

**Table 6:** Calcium content of five schemes under different soaking times.

Scheme	0 days (ppm)	1 day (ppm)	2 days (ppm)	3 days (ppm)	4 days (ppm)	5 days (ppm)
Scheme 1	18.8	22.0	24.7	25.9	27.0	27.4
Scheme 2	18.5	21.4	23.8	24.5	25.7	26.2
Scheme 3	18.4	23.4	25.4	26.8	27.8	28.4
Scheme 4	18.7	24.5	26.9	28.0	29.7	30.4
Distilled water	18.5	18.7	18.4	18.6	18.9	18.8

**Table 7:** The pH value of five schemes under different soaking times.

Scheme	0 days	1 day	2 days	3 days	4 days	5 days
Scheme 1	4.83	6.08	6.83	7.49	7.63	7.84
Scheme 2	4.92	6.25	6.97	7.71	7.74	7.83
Scheme 3	4.78	5.75	6.48	7.25	7.61	7.86
Scheme 4	4.71	5.53	6.15	7.03	7.57	7.83
Distilled water	7.94	7.89	7.95	7.87	7.86	7.85

## Data Availability

The authors confirm that the data supporting the findings of this study are available within the article.
